# SLC2A2 (GLUT2) as a novel prognostic factor for hepatocellular carcinoma

**DOI:** 10.18632/oncotarget.20266

**Published:** 2017-08-14

**Authors:** Yun Hak Kim, Dae Cheon Jeong, Kyoungjune Pak, Myoung-Eun Han, Ji-Young Kim, Liu Liangwen, Hyun Jin Kim, Tae Woo Kim, Tae Hwa Kim, Dong Woo Hyun, Sae-Ock Oh

**Affiliations:** ^1^ Department of Anatomy, School of medicine, Pusan National University, Yangsan, Republic of Korea; ^2^ Department of Statistics, Korea University, Seoul 02841, Republic of Korea; ^3^ Department of Nuclear Medicine, Pusan National University Hospital, Busan, Republic of Korea; ^4^ Eco-friendly new materials research center, Korea Research Institute of Chemical Technology (KRICT), Daejeon, Republic of Korea; ^5^ Department of Orthopaedic Surgery, Pusan National University Hospital, Yangsan, Republic of Korea; ^6^ Department of Internal Medicine, Pusan National University Hospital, Busan, Republic of Korea; ^7^ Department of General Surgery, Pusan National University Hospital, Busan, Republic of Korea

**Keywords:** hepatocellular carcinoma (HCC), solute carrier 2A (SLC2A), glucose transporter (GLUT), the cancer genome atlas (TCGA), 2-^18^fluoro-deoxy-D-glucose (^18^FDG)

## Abstract

High rates of glucose transport via solute carrier (SLC2A, GLUT) family members are required to satisfy the high metabolic demands of cancer cells, and because of this characteristic of cancer cells 2-^18^fluoro-deoxy-D-glucose (^18^FDG)-PET has become a powerful diagnostic tool. However, its sensitivity for hepatocellular carcinoma (HCC) is lower than for other malignancies, which suggests SLC2A family members are differentially expressed in HCC. In the present study, the expression patterns of SLC2A family members in tumor tissues and their associations with HCC progression were analyzed using data obtained from The Cancer Genome Atlas (TCGA). It was found that the expression of SLC2A2 (GLUT2) was higher in HCC than those of other members of the SLC2A family. The associations of the expression levels of SLC2A family members and previously known prognostic factors with clinical stages were examined using the *T*-test or the Mann-Whitney *U* test, and interestingly, SLC2A2 expression was found to be associated with an advanced clinical stage (*p* = 0.0015). Furthermore, Kaplan-Meier analysis using the log-rank or the Gehan-Breslow-Wilcoxon test showed SLC2A2 expression was positively associated with overall survival (*p* < 0.001, Gehan-Breslow-Wilcoxon test and *p* = 0.0145 by multivariate Cox regression). The prognostic significance of SLC2A2 was similar in both early and late stages. However, it was more significant in HCC patients without alcohol consumption history and hepatitis C infection. Taken together, SLC2A2 was associated with clinical stages and independently associated with overall survival in patients with HCC. We suggest that SLC2A2 be considered a new prognostic factor for HCC.

## INTRODUCTION

Cancer cells acquire energy from different various sources, including glucose and fructose, to satisfy their high metabolic demands, and glucose and related hexoses are transported into cells via glucose transporter (GLUT) family (Solute carrier, SLC2A family) [1, 2]. To date, 14 members of the GLUT family have been identified, and their expressions are known to be tissue dependent [3–8]. Glucose is transported to cancer cells by SLC2A proteins, and 2-^18^fluoro-deoxy-D-glucose (^18^FDG)-PET fundamentally relies on this process [9–12]. ^18^FDG is primarily transported to cells by SLC2A1 and/or SLC2A3, so that their expressions are the most famous and studied prognostic factors among the SLC2A family members [9, 10, 13–19]. However, the sensitivity of ^18^FDG-PET for hepatocellular carcinoma (HCC) is lower than those of other cancers [20, 21], which suggests SLC2A family members are differentially expressed in HCC.

HCC is the fifth most common cancer in men and the seventh most common in women [22, 23]. Many HCC patients have advanced disease at time of diagnosis, and this results in poor prognoses and high mortalities [22, 24]. Reported incidence rates of HCC are particularly high in East Asia, including South Korea, and in the United States the incidence HCC and the rate of HCC-associated mortality continue to increase [22, 23, 25–27]. Despite curative and palliative treatment options, survival is poor due to late diagnoses and the ability of HCC to develop chemoresistance [28, 29]. Accordingly, new diagnostic and therapeutic targets are needed to improve survival. Over recent years, many molecular targets have been identified to determine prognosis, but their supposed merits are controversial [29, 30].

In the present study, we examined mRNA expression levels of SLC2A family members in HCC because HCC showed lower sensitivity for ^18^FDG-PET, and its prognostic significance using data from The Cancer Genome Atlas (TCGA) HCC cohort [24, 31–37].

## RESULTS

### Patient characteristics

From the TCGA hepatocellular carcinoma (HCC) data, clinical data and gene expression data were analyzed (Table 1). Mean age of the 372 patients was 59.47 years, and 67.5% were males. Mean overall survival months was 26.62 months. The racial composition of the cohort was Caucasian 49.7% and Asian 42.5%. Regarding diagnoses, 97.3% had HCC, 1.9% hepatocholangiocarcinoma (mixed), and 0.8% fibrolamellar carcinoma. Stages I, II, III and IV accounted for 46.2%, 23.4%, 22.8% and 1.6%, respectively. About 75% of patients had several risk factors, and ∼25% had no primary risk factor.

**Table 1:** Patient characteristics in the TCGA cohort

			Total	%
Age (mean ± SD, *n* = 372)			59.47 ± 13.49	-
Overall survival_months (mean ± SD, *n* = 372)			26.62 ± 24.12	-
Sex (*n* = 372)	Male		251	67.5
	Female		121	32.5
AJCC stage (*n* = 372)	I		172	46.2
	II		87	23.4
	III		85	22.8
	IV		6	1.6
	Unknown		22	5.9
Race (*n* = 372)	White		185	49.7
	Asian		158	42.5
	Black or American		17	4.6
	American Indian or Alaska native		2	0.5
	Unknown		10	2.7
HistologicalDiagnosis(*n* = 372)	Hepatocellular carcinoma		362	97.3
	Hepatocholangiocarcinoma (mixed)		7	1.9
	Fibrolamellar carcinoma		3	0.8
Risk factors	Alcohol consumption	Alone	68	58.1
		Hepa B	20	17.1
		Hepa C	14	12.0
		Hepa B + C	3	2.6
		Others	12	10.3
	Hepa B	Alone	76	93.8
		Hepa C	3	3.7
		Others	2	2.5
	Hepa C	Alone	32	97.0
		Others	1	3.0
	Others alone		30	-
	No primary risk factors		91	-

### Patient selection

The total number of HCC patients in the HCC cohort was 372. Patient IDs of RNA-seq data and clinical data were matched. For two-sample location test, the exclusions were as follows (Supplementary Figure 1); [1] patients with hepatocholangiocarcinoma or fibrolamellar carcinoma (*n* = 11), [2] patients with unknown stage (*n* = 22), [3] patients with NA or –infinite (−Inf) values for each target gene (*n* = 10). For multivariate regression analysis, NA and –Inf values of all target genes were excluded at once (*n* = 16).

### Expressions of SLC2A family members and their associations with clinical stages

**Figure 1:**
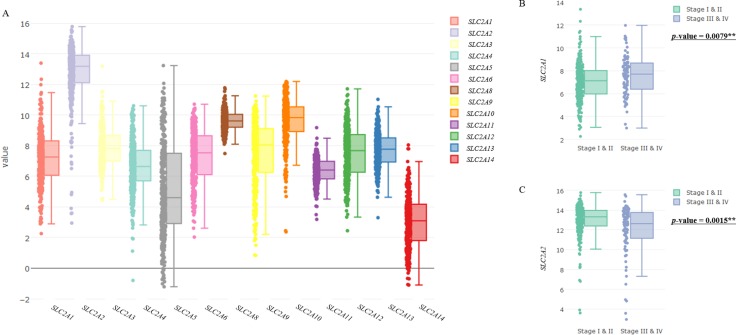
Expression levels of SLC2A family members in HCC and their association with clinical stages (**A**) Boxplots represent mRNA expressions of SLC2A family members. (**B**, **C**) Relations between clinical stages and the expression levels of SLC2A1 (B) and SLC2A2 (C) were exhibited using boxplots and scatterplots. Central lines in boxes represent medians, boxes show interquartile ranges (IQR), and error bars show the full range of values, excluding outliers defined as being more than ± 1.5 IQR outside boxes. Scatter plots represent raw data. The *p*-value in Figure 1B and 1C means results of two-sample location test of gene expression levels in Stage 1 & II vs Stage III & IV.

The mRNA expression levels of SLC2A family members in tumor tissues were analyzed using TCGA data (Figure 1A). Interestingly, the expression level of SLC2A2 (GLUT2) was higher than those of other members of the SLC2A family, and SLC2A14 was expressed the least (Figure 1A). To evaluate associations of the expression levels of SLC2A family members and previously known prognostic factors of HCC with clinical stages, we drew a box/scatter-plot (Figure 1B, 1C; Supplementary Figure 2, Figure 3) and conducted the two-sample location test (Figure 1B, 1C; Supplementary Tables 1, 2). If gene expressions were not normally distributed, we used the Mann-Whitney *U* test. If gene expressions were normally distributed and had equal variance, *T*-test was used. *T*-test with Satterthwaite approximation was used in the case of normally distributed and no equal variance. As shown in Figure 1, the expressions of SLC2A1 was found to be positively correlated with advanced stage HCC (*p* = 0.0079, Mann-Whitney *U* test), however the expression of SLC2A2 was found to be negatively associated with advanced stage (*p* = 0.0015, Mann-Whitney *U* test). To assess racial differences of SLC2A2 expression, we analyzed the mean SLC2A2 expression values. The mean SLC2A2 expression levels of each races were similar except ‘American indian or Alaska native’, because there are only 2 patients (Supplementary Table 3).

**Figure 2:**
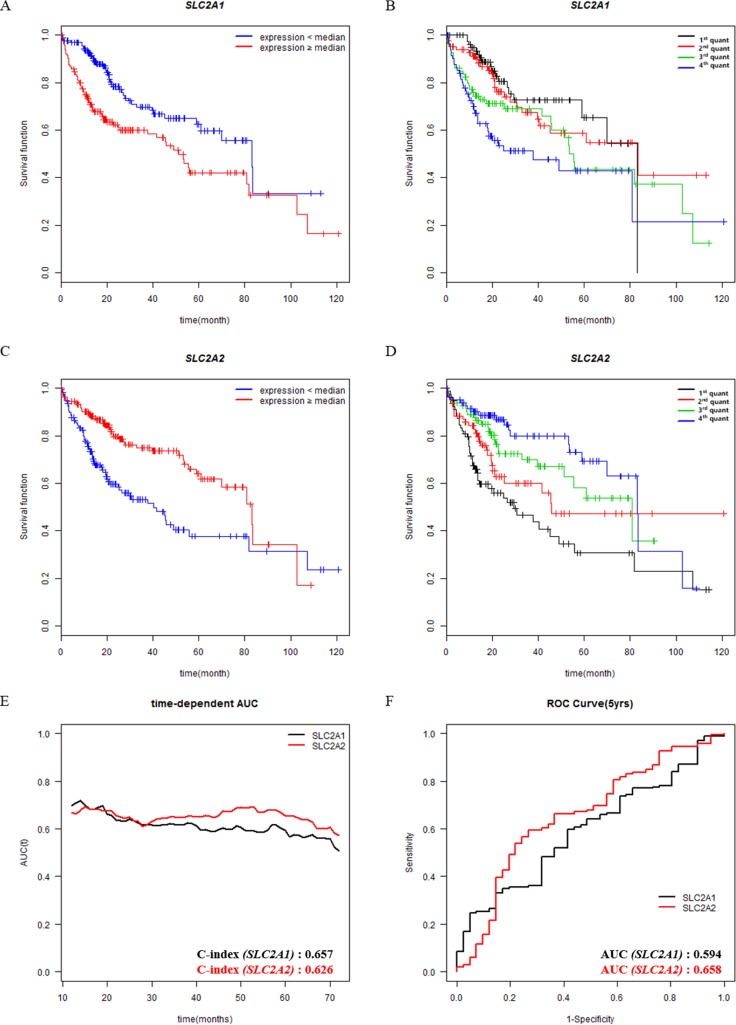
Survival analysis according to the expression levels of prognostic genes in patients with HCC (**A**, **B**, **C**, **D**) Overall survival analysis of HCC patients with respect to the expression levels of SLC2A1 or SLC2A2 was performed by Kaplan-Meier analysis. (A, C) Expression levels of genes are classified into low or high compared with the median (blue or red lines, respectively). (B, D) Expression levels of genes are classified into four from lowest quantile (1st quant) to highest quantile (4th quant). (**E**, **F**) Time-dependent Area Under the Curve (AUC) and Receiver Operating Characteristic (ROC) curve at 5 years according to the continuous expression values of SLC2A1 or SLC2A2. Both C-index and AUC value at 5 years are described at the bottom right position of E and F.

**Figure 3:**
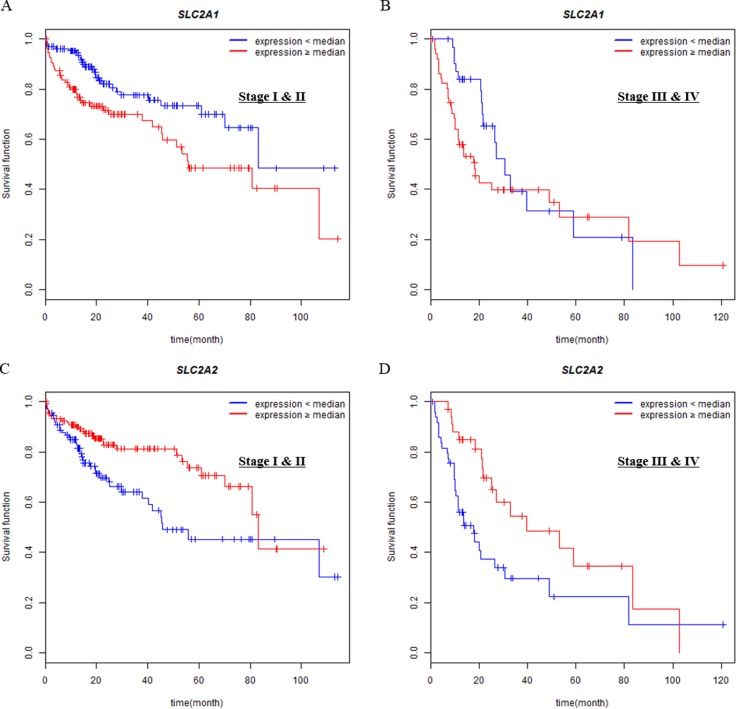
Associations of SLC2A1 or SLC2A2 expressions with overall survival in different tumor stages (I & II vs III & IV) Overall survival analysis of HCC patients with respect to the expression levels of SLC2A1 or SLC2A2 was performed by Kaplan-Meier analysis. Expression levels are classified into low or high compared with the median (blue or red lines, respectively). (**A**, **C**) The curves were analyzed in Stage I & II group. (**B**, **D**) The curves were analyzed in Stage III & IV group.

**Table 2:** Test for equality of survival distributions for different levels of gene expression

Gene name	Protein name	*p*-value	Test	Median survival (months)			
				Low expression		High expression	
*SLC2A1*	GLUT1	< 0.001***	Log-rank	**83.18**		**51.25**	
*SLC2A2*	GLUT2	< 0.001***	Gehan-breslow-Wilcoxon	**40.37**		**83.18**	
**Gene name**	**Protein name**	***p*-value**	**Test**	**Median survival (months)**			
				**1st quant**	**2nd quant**	**3rd quant**	**4th quant**
*SLC2A1*	GLUT1	< 0.001***	Gehan-breslow-Wilcoxon	**83.18**	**83.51**	**55.35**	**24.87**
*SLC2A2*	GLUT2	< 0.001***	Gehan-breslow-Wilcoxon	**29.53**	**45.89**	**80.68**	**83.18**

### Association between SLC2A family members and survival

Associations between gene expressions and patient survival were evaluated using Kaplan-Meier plots. Before the analysis, we divided the expression level of SLC2A1 or SLC2A2 into two (median expression group, Figure 2A, 2C) or four (quantile expression group, Figure 2B, 2D) groups. The Gehan-Breslow-Wilcoxon test was used for the most analysis, because survival curves were non-parallel (Figure 2B–2D and Tables 2, 3). However, the log-rank test was used for SLC2A1 (median expression group) because their survival curves were parallel (Figure 2A). Among previously known 31 prognostic genes examined, only 6 genes were found to be associated with survival and their results except SLC2A1 and SLC2A2 are shown in Supplementary Figures 4, 5 and Supplementary Tables 4, 5. In median subgroups, high expressions of SLC2A1 was associated with poor overall survival (*p* < 0.001, Figure 2A), whereas low SLC2A2 expression was associated with poor overall survival (*p* < 0.001, Figure 2C). In case of quantile subgroups, SLC2A2 expression level was associated with overall survival between each quantile groups, but SLC2A1 was not (Figure 2B, 2D and Table 2).

**Table 3:** Test for equality of survival distributions for different levels of gene expression in each risk factor group and different stage group

Gene name	Protein name	*p*-value	stage	Test	Median survival	
					Low expression	High expression
*SLC2A1*	GLUT1	0.0062**	I & II	Log rank	**83.18**	**55.68**
*SLC2A2*	GLUT2	0.0050**	I & II	Gehan-breslow-Wilcoxon	**45.89**	**83.18**
*SLC2A1*	GLUT1	0.0264*	III & IV	Gehan-breslow-Wilcoxon	**30.58**	**18.27**
*SLC2A2*	GLUT2	0.0025**	III & IV	Gehan-breslow-Wilcoxon	**17.97**	**39.75**
**Gene name**	**Protein name**	**Risk factor**	***p*-value**	**Test**	**Median survival (months)**	
					**Low expression**	**High expression**
*SLC2A2*	GLUT2	Alcohol consumption (O)	0.1675	Gehan-breslow-Wilcoxon	**30.58**	**102.66**
		Alcohol consumption (X)	< 0.001***	Gehan-breslow-Wilcoxon	**41.75**	**80.68**
		Hepatitis B (O)	0.0131*	Log rank	**NA**	**NA**
		Hepatitis B (X)	0.0083**	Gehan-breslow-Wilcoxon	**30.58**	**70.01**
		Hepatitis C (O)	0.1483	Gehan-breslow-Wilcoxon	**25.23**	**60.84**
		Hepatitis C (X)	< 0.001***	Gehan-breslow-Wilcoxon	**45.07**	**83.18**

**Table 4:** Multivariate analysis of relations between clinicopathological variables and overall survival

Full model					
Clinicopathological variables	Total *N*	Hazard Ratio	*p*-value	Lower 95%	Upper 95%
SLC2A1 (GLUT1)	339	1.0043	0.9449	0.8881	1.1359
SLC2A2 (GLUT2)		0.9096	0.0299*	0.8351	0.9908
GENDER		0.8409	0.1936	0.5745	1.2310
AGE (continuous)		1.0096	0.1936	0.9952	1.0241
STAGE (I or II vs III or IV)		2.3301	< 0.001***	1.6015	3.3918
**Selected Model (using stepwise method)**					
**Clinicopathological variables**	**Total *N***	**Hazard Ratio**	***p*-value**	**Lower 95%**	**Upper 95%**
SLC2A2 (GLUT2)	339	0.9097	0.0145*	0.8433	0.9814
AGE (continuous)		1.0105	0.1459	0.9964	1.0249
Stage (I or II vs III or IV)		2.3498	< 0.001***	1.6154	3.4182

Furthermore, median patient survival was different depending on the expression levels of genes (Table 2; Supplementary Tables 4, 5). Notably, the difference in median survival between the low and high expression groups was the widest in the SLC2A2 group. Median survival in the SLC2A2-high group was 83.18 months, whereas that in the SLC2A2-low group was 40.37 months. Median survival in the SLC2A1-high group was 51.25 months, and that in the SLC2A1-low group was 83.18 months. In addition, each quantile survival of SLC2A2 tended to be increased depending on gene expression levels (1st : 2nd : 3rd : 4th = 29.53 : 45.89 : 80.68 : 83.18), whereas quantile survival of SLC2A1 did not show the pattern (Figure 2B, 2D).

To further compare the prognostic accuracy as continuous value, we examined the C-index in time-dependent Area Under the Curve (AUC) and AUC values at 5 years for SLC2A family members or previously known prognostic genes (Figure 2E, 2F and Supplementary Tables 5, 6). Even though SLC2A1 showed a higher C-index value (0.657) than SLC2A2 (0.626), SLC2A2 has the highest AUC value (0.658) at 5 years than other genes (Figure 2E and Supplementary Tables 6, 7).

In order to identify whether the prognostic significance of SLC2A2 on survival can change depending on stages, we divided patients into two groups (Stage I & II vs Stage III & IV) and then drew Kaplan-Meier survival curve (Figure 3). The prognostic significance of SLC2A2 was similar in both early and late stages.

**Figure 4:**
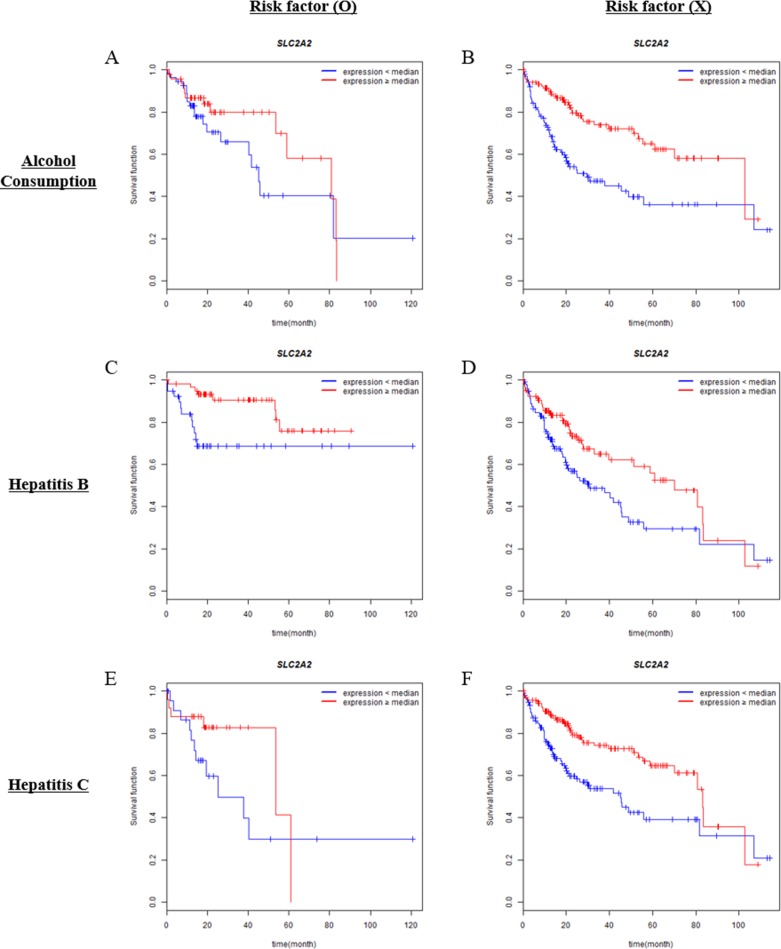
Associations of SLC2A1 or SLC2A2 expressions with overall survival in different risk factor groups Overall survival analysis of HCC patients with respect to the expression levels of SLC2A1 or SLC2A2 was performed by Kaplan-Meier analysis. Expression levels are classified into low or high compared with the median (blue or red lines, respectively). Analysis was performed in groups in the presence (**A**, **C**, **E**) or absence (**B**, **D**, **F**) of risk factors (Alcohol consumption, Hepatitis B or C infection, respectively).

There are three representative risk factors in HCC which are alcohol consumption, hepatitis B and C infection. We examined whether the presence or absence of risk factors can affect the prognostic significance of SLC2A2 expression. Interestingly, alcohol consumption history affected the prognostic significance of SLC2A2 (Figure 4A, 4B). Low SLC2A2 expression was correlated with poor overall survival in patients who had alcohol consumption history (Figure 4A, 4B and Table 3). Hepatitis B infection did not affect the prognostic significance of SLC2A2 (Figure 4C, 4D and Table 3). However, the presence or absence of hepatitis C infection affected the significance of SLC2A2. As shown in Figure 4F, in patient group who did not have hepatitis virus C infections, high SLC2A2 expression had good survival outcomes. Median survival of each group is presented in Table 3.

To assess prognostic significance of SLC2A2 depending on surgery type, we classified patients into segmentectomy or lobectomy group. Irrespective of surgery type, high SLC2A2 expression had good survival outcome in both groups (*p* = 0.07, 0.005 respectively, Supplementary Figure 6).

Since SLC2A1 and SLC2A2 are associated with survival of patients in the present study, we examined whether the combination of SLC2A2 and SLC2A1 has better prognostic significance than SLC2A2 alone. Patients were classified into 4 groups; 1) SLC2A1 < median and SLC2A2 < median, 2) SLC2A1 ≥ median and SLC2A2 < median, 3) SLC2A1 < median and SLC2A2 ≥ median, 4) SLC2A1 ≥ median and SLC2A2 ≥ median. As shown in Supplementary Figure 7, group 1 curve crossed group 2 curve from 30 to 60 months, which means the combination of SLC2A1 and SLC2A2 might not be able to discriminate prognosis of HCC patients. However, SLC2A2 alone discriminated prognosis of patients during all the period (Figure 2D).

### Multivariate analysis

Multivariate regression analysis was conducted to confirm these associations with survival. We compared SLC2A1 or SLC2A2 with other clinicopathologic variables at a time. The analysis showed that SLC2A2 expression was an independent prognostic factor for survival outcome, with a hazard ratio of 0.9097 (0.8433–0.9814, *p* = 0.0145) along with age and stage (Table 4).

## DISCUSSION

Several staging (AJCC, Okuda, BCLC) and scoring (Milan, ALBI, Child-Pugh) systems are used for hepatocellular carcinoma (HCC), but somewhat surprisingly no consensus has been reached as which best predicts survival [38–40]. Because of limitations of conventional staging systems, new molecular markers need to be identified that can be used in combination with current staging systems. The present study suggests SLC2A2 (GLUT2) has potential as a novel prognostic factor in HCC and it could be applied for the imaging of HCC.

Most cancer cells exhibit high glucose metabolism. To obtain enough energy, cancer cells up-regulate the expressions of glucose transporters (GLUTs), especially SLC2A1, which has high affinity for glucose [11, 12, 41]. SLC2A2 has relatively low affinity for glucose, mannose, galactose, and fructose, but high affinity for glucosamine [8, 42, 43]. As shown in Supplementary Table 8, different glucose transporters have different binding affinity for glucose [6, 7, 44]. 2-^18^Fluoro-deoxy-D-glucose (^18^FDG)-PET-CT provides information about the metabolic statuses of tumors. FDG is primarily transported by SLC2A1 and SLC2A3 (Supplementary Table 8) [9, 10], which suggests that FDG uptake by tumor cells will be different depending on different expression levels of glucose transporters. Notably, the sensitivity of ^18^FDG-PET for HCC is lower than for other malignant cancers [20, 21], which suggests ^18^FDG (−) HCC does not overexpress SLC2A1 and SLC2A3. In the present study, we found the expression level of SLC2A2 was higher than those of SLC2A1 or SLC2A3 in HCC (Figure 1A).

Prediction of patient's survival is very important to make decisions about therapeutic methods. According to the predicted prognosis, clinicians determined the therapeutic options among curative (resection, transplantation and ablation), palliative (transarterial chemoembolization, sorafenib), and symptomatic treatments [28, 45]. However, because there is no consensus on the use of prognostic prediction system, it is very important to develop prognostic markers. 5-year survival rate commonly used for estimating prognostic markers, because cancer-specific death usually occurs within 5 years, 25∼50% in case of total HCC [46, 47]. In the present study, we identified SLC2A2 has higher Area Under the Curve (AUC) value than other prognostic genes which suggests it may be very useful to predict 5-year survival rate of HCC.

Lower expression of SLC2A2 in HCC than in normal tissues suggests it could be used as an imaging target for diagnostic purposes [48, 49]. ^18^FDG-PET imaging is useful when cancers express high levels of SLC2A1 or SLC2A3. A specific imaging molecule targeting SLC2A2 could be used to detect HCC in a negative manner because SLC2A2 has relatively low affinity for glucose, mannose, galactose, and fructose. Furthermore, an imaging technique based on SLC2A2 could also be useful in renal cell carcinoma because SLC2A2 is mainly expressed in liver, absorptive renal cells, and pancreatic β cells [50–52].

Alcohol consumption and chronic viral hepatitis B, C infections are the major known risk factors of HCC [53–55]. In the present study, we found the prognostic value of SLC2A2 was more significant in patients who has no major risk factors (Figure 4). Although SLC2A2 is not associated with overall survival of patients with alcohol consumption or viral hepatitis C infection, the high SLC2A2 expressions seemed to be correlated with good prognosis (Figure 4). Further studies are necessary in order to conclude SLC2A2 as a prognostic marker independent of risk factors.

Altered expressions of SLC2A family members have been reported in many types of cancer (liver- SLC2A1, SLC2A2, SLC2A5; pancreas- SLC2A1; breast- SLC2A1, SLC2A2, SLC2A4*;* stomach- SLC2A2, SLC2A*4*, SLC2A*5*, SLC2A14; lymphoma- SLC2A*5*). Interestingly, overexpression of some SLC2A isoforms have been shown to be of invasiveness and poor prognosis, especially SLC2A1 [7, 13–19, 56, 57]. However, expression of SLC2A2 was inversely associated with invasiveness in breast cancer [58]. Using the HCC dataset of TCGA, we compared SLC2A2 family and known prognostic factors for associations with survival. When we looked for gene expressional differences between stages I & II vs stages III & IV, interestingly, the *p*-value of SLC2A2 was more significant than those of any other putative prognostic factors (Figure 1, Supplementary Figures 1, 2 and Supplementary Tables 2, 3). Furthermore, the association between SLC2A2 and survival was supported by the survival analysis (Figure 2 and Table 2). As shown in Figure 2B, 2D (quantile survival curve), we identified overall survival of HCC patients was highly dependent on SLC2A2 expression compared to other prognostic genes.

The present study is the first to investigate the prognostic relevance of SLC2A2 in HCC, and we believe the results warrant further studies. We suggest that it could be a target for the diagnostic imaging of HCC in addition to its prognostic significance in HCC.

## MATERIALS AND METHODS

### Patients’ data

RNA-seq expression and clinical data for hepatocellular carcinoma (HCC) were downloaded from The Cancer Genome Atlas (TCGA, last download date: 2017.4.22) and cBio Cancer Genomics Portal (last download date: 2017.4.22). This process was performed by using ‘*cgdsr’* package in R.

### Patient exclusion criteria

Forty-nine patients in HCC cohort were excluded. The reasons for exclusion were: (1) not diagnosed as HCC, (2) staging not confirmed, and (3) Not available (NA) or – infinite (−Inf) gene expression values.

### Two-sample location test

The only reason to choose one test over another in a given situation is its ability to reject a false hypothesis. The *T*-test is more powerful than the Mann-Whitney *U* test when data are normally distributed, whereas *T*-test with Satterthwaite approximation (Welch's *T*-test) is more powerful when data are normally distributed and heterogeneous. On the other hand, the *T*-test is invalid when data are not normally distributed. For this reason, we first applied the Shapiro-Wilks normality test to gene expression and then conducted the two sample location test to gene expression between different stage groups. The boxplots were drawn by using ‘*plotly’* package in R.

### Kaplan-Meier survival curves

Median values are more robust against outliers than means. For this reason, medians can be used as measures when distributions are asymmetric or when one wishes to reduce the importance of outliers. The only reason to choose one test over another in given situations is if it will be more powerful, that is, more likely to reject a false hypothesis. The log-rank test is more powerful than Gehan's Wilcoxon test for detecting departures when two survival functions are parallel. On the other hand, Gehan's Wilcoxon test appears to be more powerful than the log-rank test for detecting other types of differences, for example, when survival functions are not parallel. Survival analysis was performed by using ‘*survMisc’* and ‘*flexsurv’* packages in R.

### Discriminatory accuracy analysis

To evaluate the discriminatory accuracy as continuous value, we used UNO's C-index [59] in the time-dependent Area Under the Curve (AUC) analysis and AUC value in Receiver Operating Chracteristic (ROC) curve analysis at 5 years. These values were obtained using R package ‘*survival’* and ‘*survAUC’*.

### Multivariate Cox regression

We used multivariate cox regression to compare the effect of SLC2A1, SLC2A2 on survival along with other clinicopathological factors (AJCC stage, age, gender).

## SUPPLEMENTARY MATERIALS FIGURES AND TABLES



## Abbreviations

HCC: Hepatocellular carcinoma
SLC2A: Solute Carrier 2A
GLUT: Glucose transporter
TCGA: The Cancer Genome Atlas
SLC2A: Solute Carrier 2A
FDG: 2-^18^fluoro-deoxy-D-glucose
