# Supplementary material for: SLC2A2 (GLUT2) as a novel prognostic factor for hepatocellular carcinoma
Source: Oncotarget. 2017 Aug 14;8(40):68381–92. doi: 10.18632/oncotarget.20266 (PMC5620264; doi:10.18632/oncotarget.20266)
Supplement: Supplementary file 1 [file oncotarget-08-68381-s001.pdf]

SLC2A2 (GLUT2) as a novel prognostic factor for hepatocellular carcinoma

SUPPLEMENTARY MATERIALS

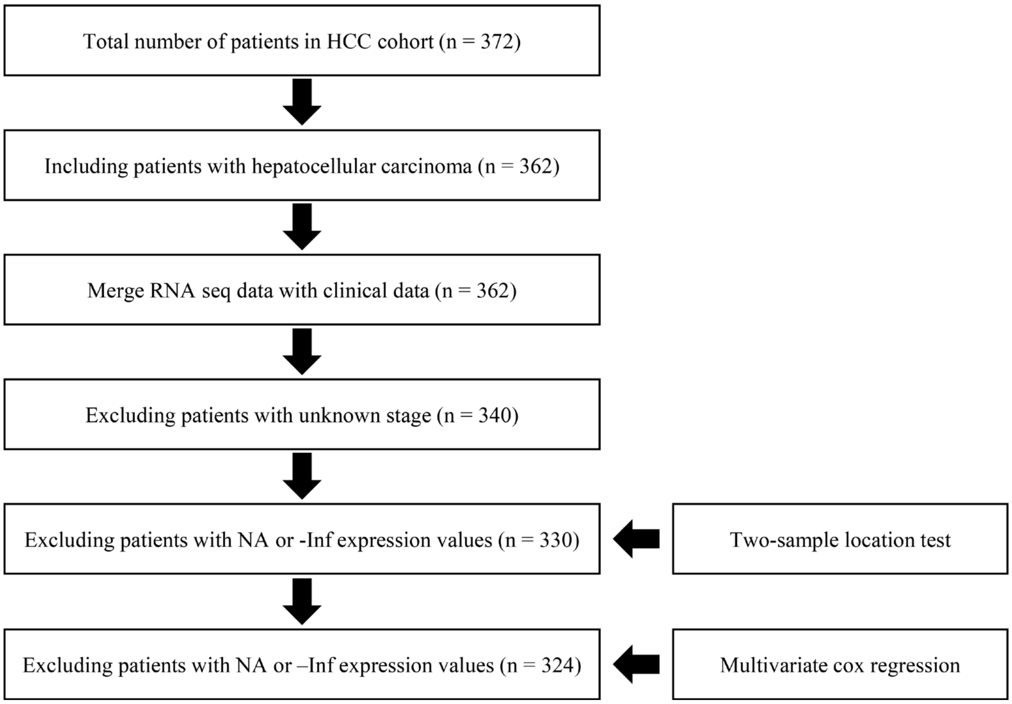

Supplementary Figure 1: Flow diagram of patient inclusion/exclusion criteria.

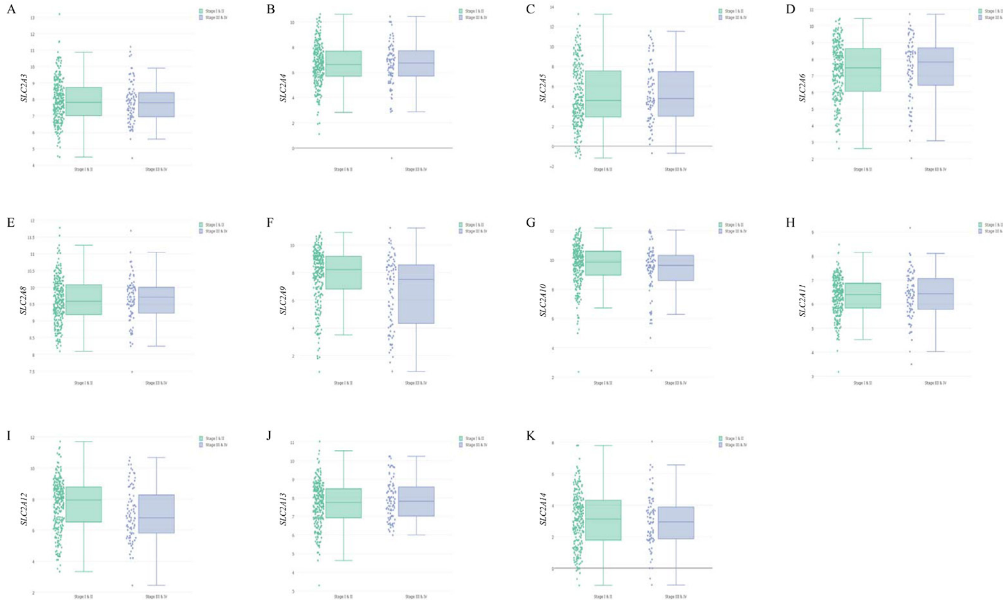

Supplementary Figure 2: Association of gene expression (SLC2A family) with clinical stages. Relations between clinical stages and the expression levels of genes were examined using boxplots and scatterplots. Central lines in boxes represent medians, boxes show interquartile ranges (IQR), and error bars show the full range of values, excluding outliers defined as being more than ± 1.5IQR outside boxes. Scatter plots represent raw data.

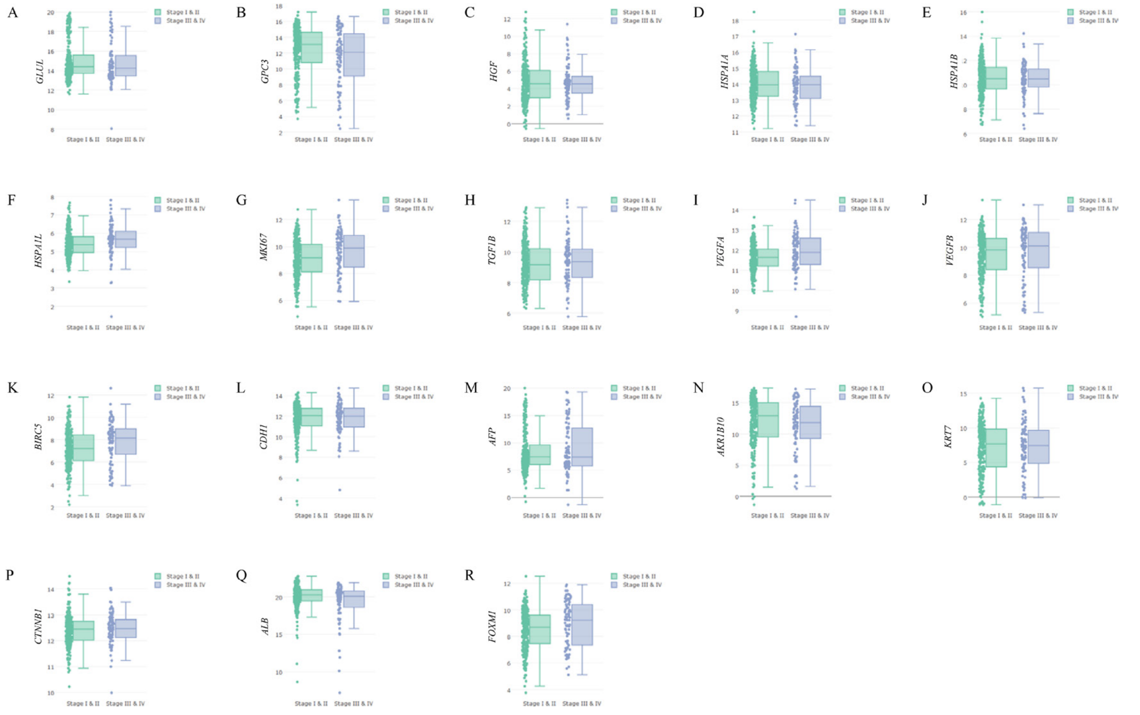

Supplementary Figure 3: Association of gene expression (previously known prognostic genes) with clinical stages. Relations between clinical stage and the expression levels of genes were examined using boxplots and scatterplots. Central lines in boxes represent medians, boxes show interquartile ranges (IQR), and error bars show the full range of values, excluding outliers defined as being more than ± 1.5IQR outside boxes. Scatter plots represent raw data.

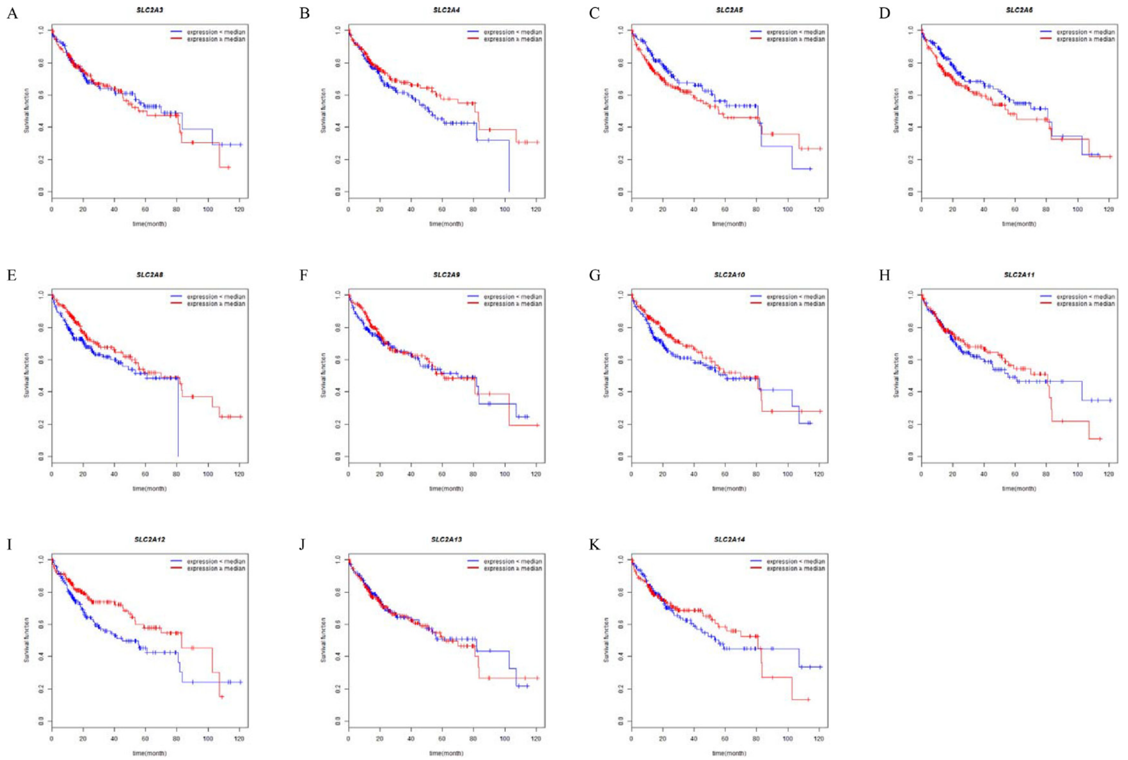

Supplementary Figure 4: Kaplan-Meier survival curves of HCC patients stratified by expression levels of SLC2A family genes. Overall survival analysis of HCC patients with respect to the expression levels of different genes was performed by Kaplan-Meier analysis. Expression levels are divided into low or high compared with the median (blue or red lines, respectively).

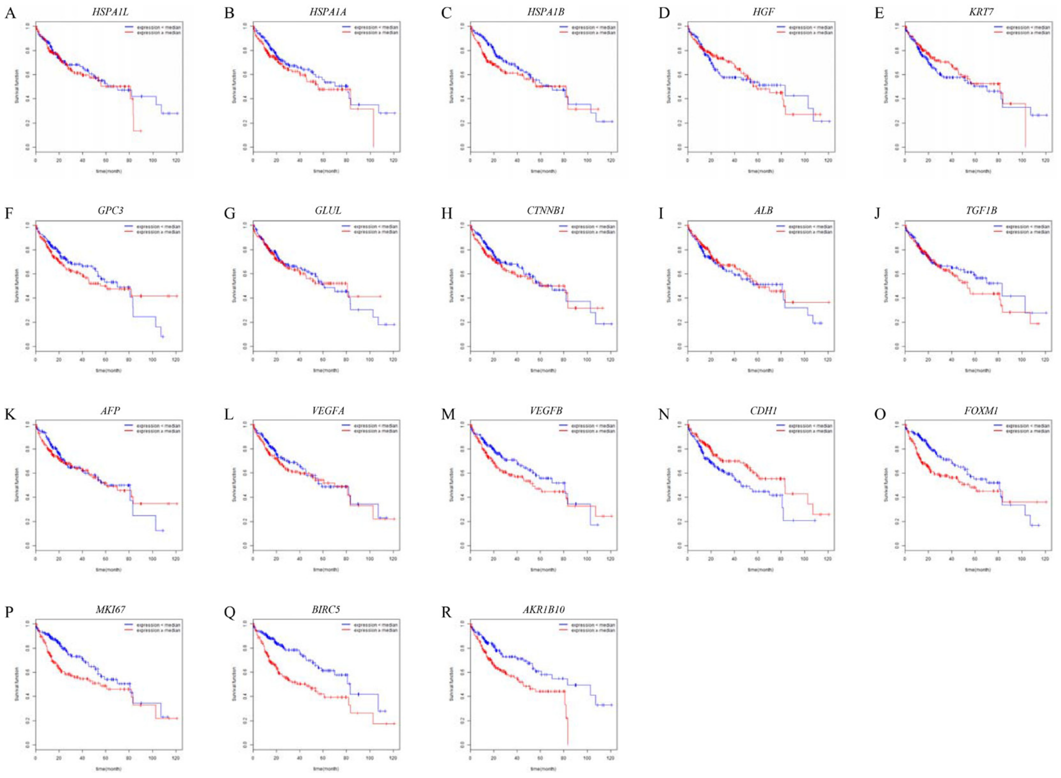

Supplementary Figure 5: Kaplan-Meier survival curves of HCC patients stratified by expression levels of previously known prognostic genes. Overall survival analysis of HCC patients with respect to the expression levels of different genes was performed by Kaplan-Meier analysis. Expression levels are divided into low or high compared with the median (blue or red lines, respectively).

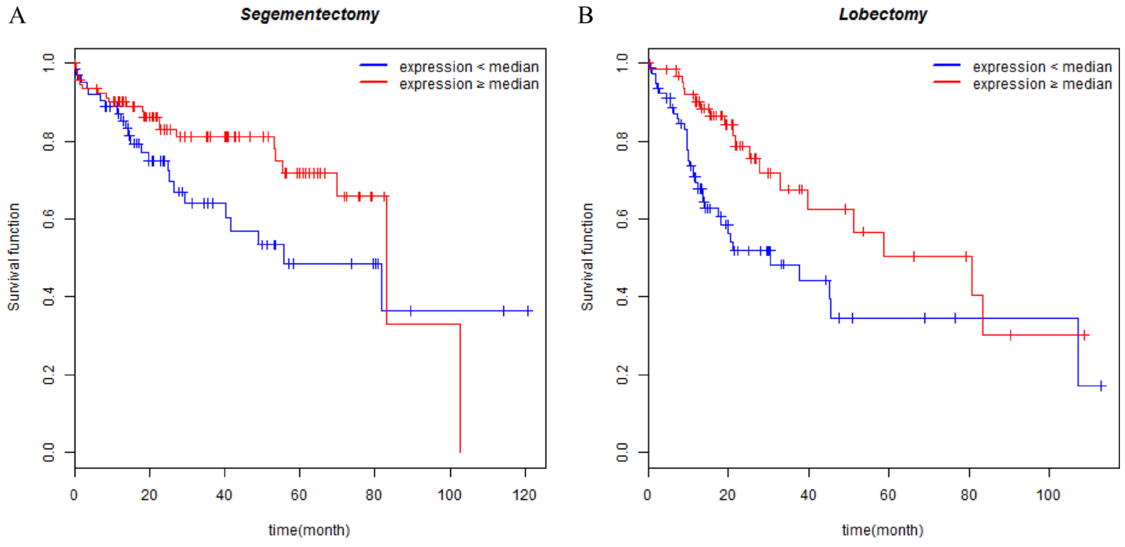

Supplementary Figure 6: Kaplan-Meier survival curves of HCC patients stratified by expression levels of SLC2A2 in different surgery type (A) Segmentectomy (B) Lobectomy.

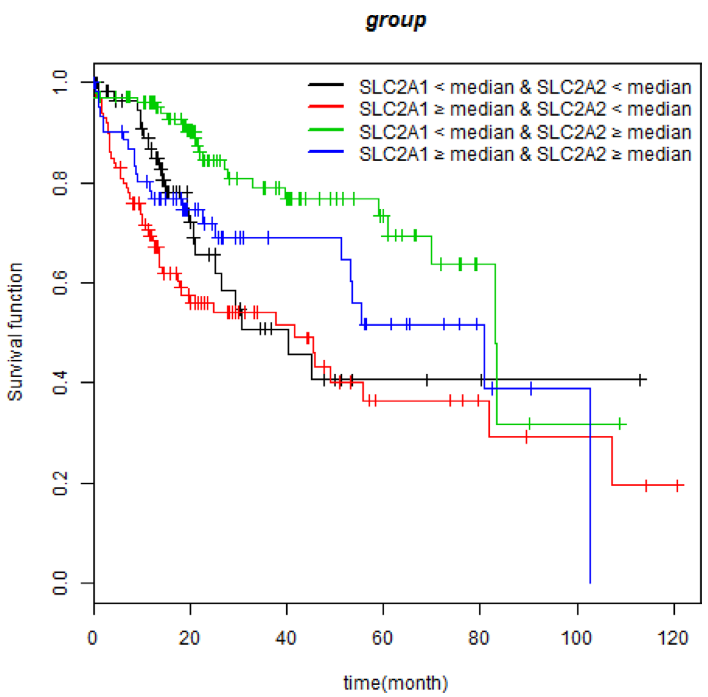

Supplementary Figure 7: Kaplan-Meier survival curves of HCC patients stratified by expression levels of SLC2A1 with SLC2A2.

Supplementary Table 1: Two-sample location test results comparing stage 1 & II and stage III & IV on SLC2A family gene expression

Gene name	Protein name	*p*-value	Test
SLC2A3	Glucose transporter 3	0.5598	Mann-Whitney U
SLC2A4	Glucose transporter 4	0.9914	Mann-Whitney U
SLC2A5	Glucose transporter 5	0.5582	Mann-Whitney U
SLC2A6	Glucose transporter 6	0.5048	Mann-Whitney U
SLC2A8	Glucose transporter 8	0.6690	Mann-Whitney U
SLC2A9	Glucose transporter 9	< 0.001***	Mann-Whitney U
SLC2A10	Glucose transporter 10	0.0666	Mann-Whitney U
SLC2A11	Glucose transporter 11	0.5943	Mann-Whitney U
SLC2A12	Glucose transporter 12	0.0037**	Mann-Whitney U
SLC2A13	Glucose transporter 13	0.3856	Mann-Whitney U
SLC2A14	Glucose transporter 14	0.5112	Mann-Whitney U

Supplementary Table 2: Two-sample location test results comparing stage 1 & II and stage III & IV on known prognostic gene expression

Gene name	Protein name	*p*-value	Test
GLUL	Glutamate synthetase	0.1538	Mann-Whitney U
GPC3	Glypican 3	0.0513	Mann-Whitney U
HGF	Hepatocyte growth factor	0.9271	Mann-Whitney U
HSPA1A	Heat shock protein 70	0.3292	*T*-test
HSPA1B	Heat shock protein 70	0.8217	Mann-Whitney U
HSPA1L	Heat shock protein 70	0.0066**	Mann-Whitney U
MKI67	Ki67	0.0093**	Mann-Whitney U
TGF1B	Transforming growth factor-β	0.5530	*T*-test
VEGFA	Vascular endothelial growth factor A	0.0054**	Welch's t-test
VEGFB	Vascular endothelial growth factor B	0.2145	Mann-Whitney U
BIRC5	Survivin	0.0072**	Mann-Whitney U
CDH1	E-cadherin	0.9687	Mann-Whitney U
AFP	Alpha-fetoprotein	0.7938	Mann-Whitney U
AKR1B10	Aldo-keto reductase family 1 B10	0.2571	Mann-Whitney U
KRT7	Cytokeratin-7	0.8784	Mann-Whitney U
CTNNB1	β-catenin	0.3805	Mann-Whitney U
ALB	Albumin	0.0528	Mann-Whitney U
FOXM1	Forkhead box protein M1	0.0299*	Mann-Whitney U

Supplementary Table 3: Comparison of SLC2A2 expression according to races

Race	Total	*SLC2A2* expression (mean ± SD)
White	185	12.80 ± 1.93
Asian	158	12.48 ± 2.39
Black or American	17	12.85 ± 0.95
American Indian or Alaska native	2	10.22 ± 4..84
Unknown	10	12.76 ± 1.60

Supplementary Table 4: Test for equality of survival distributions for the different levels of SLC2A family gene expression

Gene name	Protein name	Significance	Median survival (months)	
			Low expression	High expression
SLC2A3	Glucose transporter 3	No significance	**70.01**	**55.35**
SLC2A4	Glucose transporter 4	No significance	**51.25**	**80.68**
SLC2A5	Glucose transporter 5	No significance	**80.68**	**55.65**
SLC2A6	Glucose transporter 6	No significance	**80.68**	**55.35**
SLC2A8	Glucose transporter 8	No significance	**60.84**	**70.01**
SLC2A9	Glucose transporter 9	No significance	**70.01**	**60.84**
SLC2A10	Glucose transporter 10	No significance	**60.84**	**70.01**
SLC2A11	Glucose transporter 11	No significance	**60.84**	**70.01**
SLC2A12	Glucose transporter 12	No significance	**55.35**	**83.18**
SLC2A13	Glucose transporter 13	No significance	**81.67**	**60.84**
SLC2A14	Glucose transporter 14	No significance	**53.29**	**80.68**

Supplementary Table 5: Test for equality of survival distributions for the different levels of previously known prognostic gene expression

Gene name	Protein name	Significance (*p*-value)	Median survival (months)	
			Low expression	High expression
GLUL	Glutamate synthetase	No significance	**60.84**	**80.68**
GPC3	Glypican 3	No significance	**70.01**	**55.35**
HGF	Hepatocyte growth factor	No significance	**83.18**	**55.65**
HSPA1A	Heat shock protein 70	No significance	**70.01**	**55.35**
HSPA1B	Heat shock protein 70	No significance	**70.01**	**55.65**
HSPA1L	Heat shock protein 70	No significance	**60.84**	**80.68**
MKI67	Ki67	Significance (0.0027**)	**70.01**	**55.35**
TGF1B	Transforming growth factor-β	No significance	**83.18**	**53.35**
VEGFA	Vascular endothelial growth factor A	No significance	**58.84**	**70.01**
VEGFB	Vascular endothelial growth factor B	No significance	**80.68**	**53.29**
BIRC5	Survivin	Significance (< 0.001***)	**83.18**	**37.75**
CDH1	E-cadherin	No significance	**53.29**	**83.18**
AFP	Alpha-fetoprotein	No significance	**58.84**	**60.84**
AKR1B10	Aldo-keto reductase family 1 B10	Significance (< 0.001***)	**83.18**	**45.07**
KRT7	Cytokeratin-7	No significance	**58.84**	**80.68**
CTNNB1	β-catenin	No significance	**70.01**	**60.84**
ALB	Albumin	No significance	**80.68**	**60.84**
FOXM1	Forkhead box protein M1	Significance (< 0.001***)	**80.68**	**48.95**

Supplementary Table 6: C-index and Area Under the Curve (AUC) at 5 years of SLC2A family gene expression

Gene name	Protein name	C-index	AUC at 5 years
SLC2A3	Glucose transporter 3	0.503	0.499
SLC2A4	Glucose transporter 4	0.580	0.657
SLC2A5	Glucose transporter 5	0.608	0.567
SLC2A6	Glucose transporter 6	0.543	0.588
SLC2A8	Glucose transporter 8	0.562	0.515
SLC2A9	Glucose transporter 9	0.531	0.479
SLC2A10	Glucose transporter 10	0.553	0.522
SLC2A11	Glucose transporter 11	0.520	0.590
SLC2A12	Glucose transporter 12	0.519	0.548
SLC2A13	Glucose transporter 13	0.522	0.522
SLC2A14	Glucose transporter 14	0.508	0.562

Supplementary Table 7: C-index and Area Under the Curve (AUC) at 5 years of known prognostic gene expression

Gene name	Protein name	C-index	AUC at 5 years
GLUL	Glutamate synthetase	0.467	0.555
GPC3	Glypican 3	0.520	0.524
HGF	Hepatocyte growth factor	0.525	0.490
HSPA1A	Heat shock protein 70	0.536	0.542
HSPA1B	Heat shock protein 70	0.545	0.541
HSPA1L	Heat shock protein 70	0.521	0.504
MKI67	Ki67	0.619	0.582
TGF1B	Transforming growth factor-β	0.541	0.585
VEGFA	Vascular endothelial growth factor A	0.546	0.512
VEGFB	Vascular endothelial growth factor B	0.512	0.528
BIRC5	Survivin	0.629	0.623
CDH1	E-cadherin	0.584	0.558
AFP	Alpha-fetoprotein	0.555	0.511
AKR1B10	Aldo-keto reductase family 1 B10	0.584	0.599
KRT7	Cytokeratin-7	0.544	0.519
CTNNB1	β-catenin	0.554	0.417
ALB	Albumin	0.575	0.516
FOXM1	Forkhead box protein M1	0.622	0.600

Supplementary Table 8: Tissue distribution and substrates binding affinity of GLUT isoforms

Gene name	Protein name	Major site expression	High binding affinity
SLC2A1	GLUT 1	Ubiquitous	Glucose, FDG
SLC2A2	GLUT 2	Liver, islets, kidney, small intestine	Glucosamine
SLC2A3	GLUT 3	Brain, nerve cells	Glucose, FDG
SLC2A4	GLUT 4	Muscle, fat, heart	Glucose, glucosamine
SLC2A5	GLUT 5	Intestine, kidney, testis	Fructose
SLC2A6	GLUT 6	Spleen, leukocytes, brain	Cytochalasin B
SLC2A7	GLUT7	Small intestine, colon, testis	Fructose
SLC2A8	GLUT 8	Testis, brain, muscle, adipocyte, blastocyst	Glucose
SLC2A9	GLUT 9	Liver, kidney	-
SLC2A10	GLUT 10	Liver, pancreas	2-Deoxy glucose
SLC2A11	GLUT 11	Heart, muscle	-
SLC2A12	GLUT 12	Heart, prostate, mammary gland	-
SLC2A13	GLUT 13	Brain	*myo*-inositol
